# Intra-Areal Visual Topography in Primate Brains Mapped with Probabilistic Tractography of Diffusion-Weighted Imaging

**DOI:** 10.1093/cercor/bhab364

**Published:** 2021-11-03

**Authors:** K Tang-Wright, J E T Smith, H Bridge, K L Miller, T B Dyrby, B Ahmed, N L Reislev, J Sallet, A J Parker, K Krug

**Affiliations:** Department of Physiology Anatomy and Genetics, University of Oxford, Oxford, OX1 3PT, UK; Department of Physiology Anatomy and Genetics, University of Oxford, Oxford, OX1 3PT, UK; Ernst Strüngmann Institute (ESI) for Neuroscience in cooperation with Max Planck Society, 60528 Frankfurt, Germany; Wellcome Centre for Integrative Neuroimaging, FMRIB, Nuffield Department of Clinical Neurosciences, University of Oxford, Oxford OX3 9DU, UK; Wellcome Centre for Integrative Neuroimaging, FMRIB, Nuffield Department of Clinical Neurosciences, University of Oxford, Oxford OX3 9DU, UK; Danish Research Centre for Magnetic Resonance, Centre for Functional and Diagnostic Imaging and Research, Copenhagen University Hospital, Amager & Hvidovre, 2650 Hvidovre, Denmark; Department of Applied Mathematics and Computer Science, Technical University of Denmark, 2800 Kongens Lyngby, Denmark; Department of Physiology Anatomy and Genetics, University of Oxford, Oxford, OX1 3PT, UK; Danish Research Centre for Magnetic Resonance, Centre for Functional and Diagnostic Imaging and Research, Copenhagen University Hospital, Amager & Hvidovre, 2650 Hvidovre, Denmark; Wellcome Centre for Integrative Neuroimaging, Department of Experimental Psychology, University of Oxford, Oxford OX1 3UD, UK; Université Lyon 1, INSERM, Stem Cell and Brain Research Institute U1208, 69500 Bron, France; Department of Physiology Anatomy and Genetics, University of Oxford, Oxford, OX1 3PT, UK; Institute of Biology, Otto-von-Guericke-University Magdeburg, 39120 Magdeburg, Germany; Leibniz Institute for Neurobiology, 39118 Magdeburg, Germany; Department of Physiology Anatomy and Genetics, University of Oxford, Oxford, OX1 3PT, UK; Institute of Biology, Otto-von-Guericke-University Magdeburg, 39120 Magdeburg, Germany; Leibniz Institute for Neurobiology, 39118 Magdeburg, Germany; Centre for Behavioral Brain Sciences, Otto-von-Guericke-University Magdeburg, 39106 Magdeburg, Germany

**Keywords:** LGN, macaque, pathways, retinotopy, visual cortex

## Abstract

Noninvasive diffusion-weighted magnetic resonance imaging (dMRI) can be used to map the neural connectivity between distinct areas in the intact brain, but the standard resolution achieved fundamentally limits the sensitivity of such maps. We investigated the sensitivity and specificity of high-resolution postmortem dMRI and probabilistic tractography in rhesus macaque brains to produce retinotopic maps of the lateral geniculate nucleus (LGN) and extrastriate cortical visual area V5/MT based on their topographic connections with the previously established functional retinotopic map of primary visual cortex (V1). We also replicated the differential connectivity of magnocellular and parvocellular LGN compartments with V1 across visual field positions. Predicted topographic maps based on dMRI data largely matched the established retinotopy of both LGN and V5/MT. Furthermore, tractography based on in vivo dMRI data from the same macaque brains acquired at standard field strength (3T) yielded comparable topographic maps in many cases. We conclude that tractography based on dMRI is sensitive enough to reveal the intrinsic organization of ordered connections between topographically organized neural structures and their resultant functional organization.

## Introduction

Topographic maps are a fundamental feature of structures throughout the brain in both sensory and motor systems. This ordered representation allows for efficient encoding of highly correlated information across nearby neurons and is the substrate for information transfer between brain areas (Sperry 1961; Barlow 1986; Falchier et al. 2002; Jbabdi, Sotiropoulos, et al. 2013). In the visual system, this topography takes the form of retinotopic maps which convey spatially ordered information from the retina to the brain. These maps are maintained throughout the visual system across many levels of visual processing (Holmes 1918; Talbot and Marshall 1941; Daniel and Whitteridge 1961; Malpeli and Baker 1975; Zeki 1976; Connolly and Van Essen 1984; Merigan and Maunsell 1990; Felleman and Van Essen 1991; Nealey and Maunsell 1994; Malpeli et al. 1996; Gur et al. 1997), and therefore, require precise white matter connectivity between regions. While retinotopic maps were established initially with invasive methods, such as study of brain injury, histology, and electrophysiology, they have been visible using functional magnetic resonance imaging and retinotopic mapping for almost 30 years (e.g., Wandell and Winawer 2011). However, the precise white matter connectivity has not been demonstrated.

Diffusion-weighted magnetic resonance imaging (dMRI) is a noninvasive, indirect measure of structural brain connectivity based on the most probable diffusion patterns of water molecules with fundamental statistical and methodological limitations (Basser et al. 2000; Jones 2010; Jones et al. 2013). Tractography based on dMRI (Mori et al. 1999; Behrens, Woolrich, et al. 2003) allows the noninvasive mapping of area-to-area connections and the trajectories of larger fiber bundles (Catani and Thiebaut de Schotten 2008; Donahue et al. 2016). This approach has also been used (i) to parcellate cortex into distinct areas, based on their connectivity profiles (Johansen-Berg et al. 2004; Rushworth et al. 2006), (ii) to conduct cross-species comparisons (Hofer et al. 2008; Rilling et al. 2008; Mars et al. 2015; Balezeau et al. 2020; Barrett et al. 2020; Roumazeilles et al. 2020) and (iii) to predict functional activations in target structures (Grotheer et al. 2020). When compared with histological data, probabilistic tractography can map area-to-area connectivity well, although not without errors (Thomas et al. 2014; Azadbakht et al. 2015; Donahue et al. 2016; Maier-Hein et al. 2017; Ambrosen et al. 2020; Girard et al. 2020). Furthermore, tractography can identify functionally relevant subdivisions of the thalamic gray matter from the different profiles of their thalamocortical connections (Behrens, Johansen-Berg, et al. 2003) and the visual functional organization of the corpus callosum from interhemispheric connections (Dougherty et al. 2005; Saenz and Fine 2010).

While cortical areas, or subcortical nuclei, with different functions can be delineated by their distinct connectivity using dMRI and tractography, here, we take a further step in testing the resolving power of dMRI and tractography by delineating functional topography “within” a distinct functional thalamic nucleus and within a cortical area based solely on brain connectivity. The retinotopy of the primate visual system with its fine-grained organization and relatively large size of visual cortex is an excellent system for examining the resolution limits of imaging methods (Large et al. 2016) which can capture the 3D trajectory of an entire visual pathway. The ability of probabilistic tractography from dMRI data to reveal retinotopically ordered connectivity has been shown in humans within the visual part of the corpus callosum and for the visual hemifield maps in V1 and V2, which are directly juxta-positioned across the visual map reversal (Saenz and Fine 2010; Attar et al. 2020).

Here, we use tractography to map the visual topographic pattern of connections in the rhesus macaque (*Macaca mulatta*) between distinct subcortical and cortical structures of the visual system. In the rhesus macaque, the established functional retinotopic maps of the lateral geniculate nucleus (LGN) and primary visual cortex (V1) are reliably orientated and positioned with regard to structural landmarks (Van Essen et al. 1984; Erwin et al. 1999). Furthermore, within the LGN–V1 projection, Malpeli et al. (1996) (see also Azzopardi et al. 1999) proposed that the functionally distinct magnocellular and parvocellular pathways from LGN to V1 have different distributions of afferent fibers: Parvocellular afferents are almost uniformly distributed throughout V1, while the overall smaller number of magnocellular afferents increases in density with visual eccentricity. This manifests in an increasing magnocellular/parvocellular afferent density ratio with increasing visual eccentricity (Connolly and Van Essen 1984; Schein and de Monasterio 1987; Malpeli et al. 1996). In turn, an extensively studied target of V1 projections is the extrastriate visual area V5/MT, which is located in the posterior bank of the superior temporal sulcus (STS). V5/MT is important for visual motion and depth processing and for perceptual decision-making, and it also has a well-established retinotopic map (Zeki 1974; Van Essen et al. 1981; Salzman et al. 1990; Movshon and Newsome 1996; Krug et al. 2013; Krug 2020).

dMRI images were obtained in vivo in a clinical grade 3T scanner. For postmortem scanning, a dedicated 4.7T research magnetic resonance imaging (MRI) scanner was used to provide higher image quality and resolution compared with in vivo scans (Dyrby et al. 2011; Dyrby et al. 2018) from the same rhesus macaque brains. Using probabilistic tractography, we predicted maps of visual eccentricity and elevation within LGN from estimates of its connections to different subregions of V1 and compared those quantitatively to the established functional maps. We also estimated the relative strength of the magnocellular and parvocellular contributions to the LGN–V1 projection. Extending this approach to cortico-cortical connections, we predicted the visual eccentricity maps of cortical area V5/MT from its connections with V1. Topographic maps predicted from postmortem dMRI and tractography matched established retinotopy well. In many cases, there was also concordance of the topographic maps from in vivo and postmortem dMRI. Thus, tractography is potentially specific enough to resolve key aspects of the intra-area topographic organization of thalamic nuclei and cortical areas alike.

## Materials and Methods

### Animals

Twelve cortical hemispheres from six adult macaques (*M. mulatta*) were included in this study—three females (M124, M128, and M129) and three males (M126, M127, and M130). Mean age at death for postmortem scanning was 7.8 years (range = 3.9–12.4 years, standard deviation [SD] = ±4.0 years, weight range = 5.20–14.07 kg). All animal procedures were carried out in accordance with Home Office (UK) Regulations and European Union guidelines (EU Directive 86/609/EEC; EU Directive 2010/63/EU).

### Anesthesia

In vivo data under anesthesia were acquired from five of the rhesus macaques in this study (M124, M126, M127, M128, and M129). Anesthesia was induced using intramuscular injections of ketamine (10 mg/kg), xylazine (0.125–0.25 mg/kg), and midazolam (0.1 mg/kg). Injections of atropine (0.05 mg/kg i.m.) and meloxicam (0.2 mg/kg i.v.) were given. Local anesthetics (5% lidocaine/prilocaine cream topically applied to the outer ear canal, in some experiments, 2.5% bupivacaine injected subcutaneously around the ears) were also administered at least 15 min before scanning to prevent stimulation by the stereotactic head frame in which animals were placed. During scanning, anesthesia was maintained using sevoflurane (2–3% by inhalation), and animals were ventilated with intermittent positive pressure during the scanning session. Respiration rate, inspired-expired CO_2_, inspired-expired sevoflurane concentration, heart rate, core temperature, blood pressure, and blood oxygen saturation (SpO_2_) were monitored throughout.

### Perfusion, Tissue Fixation, and Storage

Animals were sedated with an intramuscular injection of ketamine (20 mg/kg), given an intravenous injection of Euthatal (pentobarbitone) (65 mg/kg), and were perfusion-fixated transcardially using phosphate-buffered saline (PBS), followed by 4% paraformaldehyde (PFA) in 0.1 mol/l PBS (pH 7.4) (see also Ahmed et al. 2012). Brains were removed and were stored in 4% PFA at 4°C. Preparation for postmortem imaging followed the setup described in Dyrby et al. (2011). In brief, 1 week prior to scanning, the PFA was washed out with PBS to increase T2 contrast (Thelwall et al. 2006; Leprince et al. 2015). Before scanning, the brain was sealed in a plastic bag with minimal surrounding PBS to avoid tissue dehydration. It was kept overnight at room temperature for temperature stabilization.

### Data Acquisition

In vivo data were acquired with a 3T clinical MRI scanner (full size, horizontal bore as standardly used in clinical settings) with a four-channel phased-array coil (Windmiller Kolster Scientific) and a twice-refocused spin-echo sequence (Reese et al. 2003). During the scan, the anesthetized animal’s head was secured in a stereotactic frame (Crist Instrument Company). The dMRI dataset included three *b* = 0 s/mm^2^ and single shell with 61 gradient directions using *b* = 1000 s/mm^2^. Whole-brain dMRI volumes were collected at 1 × 1 × 1 mm resolution (field of view FoV = 96 × 96 mm, image matrix = 96 × 96 for females; FoV = 112 × 112 mm, image matrix = 112 × 112 for males) as 56 interleaved axial slices using repetition time (TR) = 10 000 ms and echo time (TE) = 103 ms. The FoV was enlarged for males to accommodate their larger heads and to minimize artifacts like wrap-around.

Each 61-direction, diffusion-weighted imaging (dMRI) scan took 13 min. To minimize susceptibility-induced geometric distortions due to magnetic field inhomogeneity and to improve the signal-to-noise ratio (SNR), we collected 12 scan runs of diffusion-weighted data: 6 with one phase encode direction and interleaved with those, 6 where the phase encode direction was reversed (right-left and left-right). This method exploits the fact that images with opposite polarities show opposite distortions (Andersson et al. 2003). In total, at least 12 dMRI scan runs were collected for each animal in vivo. For some animals, we collected additional dMRI scan runs on other days.

For each animal, five high-resolution (0.5 × 0.5 × 0.5 mm) T1-weighted structural images were also acquired using a 3D magnetization-prepared rapid-acquisition gradient echo (MPRAGE) sequence with the following parameters: 128 interleaved sagittal slices (no gap), 0.5 mm slice thickness, TR = 2500 ms, TE = 4 ms, TI = 1100 ms, flip angle = 7°, FoV = 128 × 128 mm, image matrix = 256 × 256. The total scan time for the structural and diffusion weighted protocols combined was approximately 4 h.

Postmortem data were acquired with an experimental 4.7T Agilent preclinical MR scanner with a maximum gradient strength of 600 mT/m. Diffusion-weighted imaging (dMRI) was collected using a quadrature volume primate head radio frequency coil and a 2D single spin-echo sequence with single-line read-out. To minimize short-term instabilities in the postmortem dMRI data (i.e., nonlinear motion artifacts) due to the physical handling of the tissue, a dummy scan of at least 4 h was acquired prior to the actual dMRI dataset acquisition. A temperature-controlled airflow at room temperature surrounded the tissue during the scanning session (for see details, see Dyrby et al. 2011; Dyrby et al. 2018). The dMRI dataset included three *b* = 0 s/mm^2^ and single shell with 61 gradient directions (Jones 2004) using *b* = 4310 s/mm^2^ (Gradient strength, G = 100 mT/m, δ = 27 ms, and Δ = 33.5 ms). Whole-brain dMRI volumes were collected at 0.5 × 0.5 × 0.5 mm resolution (FoV = 64 × 128 mm, image matrix = 128 × 256) as 128 interleaved axial slices using TR = 5100 ms and TE = 45 ms. Scan time for each dMRI dataset was 12 h and was repeated four times, giving an approximate total scan time of 48 h for each macaque brain (excluding the dummy scan). We have not observed, by visual inspection, any effect of b0 drift when overlaying non-DWI images acquired at different time points and no image registration was needed (Dyrby et al. 2011).

Compared to the in vivo dMRI scans, the postmortem data were acquired with a higher *b*-value (*b* = 4310 s/mm^2^ vs. *b* = 1000 s/mm^2^) to account for the lower diffusivity in postmortem tissue and had an 8× higher resolution (0.125 mm^3^ vs. 1 mm^3^), and of course, no physiological noise (Sun et al. 2005; Dyrby et al. 2011; Walker et al. 2011; Dyrby et al. 2018).

### In Vivo Data Preprocessing

Brain masks were created for the in vivo diffusion-weighted images using a combination of thresholding the b0-averaged image and subsequent manual corrections. To reduce the distortions from magnetic field inhomogeneities, the two sets of dMRI with opposite phase encoding polarity were combined using FSL’s “TopUp” tool (Andersson et al. 2003) (FMRIB Software Library (FSL); www.fmrib.ox.ac.uk/fsl; Smith et al. 2004; Woolrich et al. 2009; Jenkinson et al. 2012). Distortions from eddy currents in the gradient coils were reduced both through the use of the twice-refocused dMRI sequence (Reese et al. 2003) and the FSL eddy-correction software (using “eddy”). After correction, data were checked visually by looping through all the volumes with the movie-mode in FSLview. In a final step, individual gradient images were first prepared from the two phase-encoding directions separately; then, images with the same gradient but with different phase encoding directions were averaged to improve SNR. After preprocessing, image quality was checked by measuring the average fractional anisotropy (FA) of voxels containing the corpus callosum in a parasagittal slice midway between the two hemispheres for each brain. One brain (M126) was excluded from further analysis based on an inclusion criterion of FA ≥ 0.5. The FA from the excluded brain was 0.47 (SD = 0.18), whereas the average FA for the included brains was 0.60 (SD = 0.16). This excluded brain also had considerable signal dropout in the occipital lobe. The scans included in the analysis also showed stable SNR values over time in measures (signal over SD) taken from the b0 images with a corpus callosum mask. For two brains (M127 and M128), we obtained two good sets of 12 dMRI scan runs taken on different days, which were combined after standard preprocessing of each set of scan runs obtained on a single day using linear registration (FSL, FLIRT). In this case, the two sets of scans for each brain were then averaged together following registration for a total of 24 averaged repeats.

### Postmortem Data Preprocessing

Visual inspection of the acquired postmortem dMRI dataset confirmed no motion, scanner drifts, or other artifacts which often affect image quality in vivo and would require correction. Therefore, the four repeat acquisitions were averaged off-line for each brain. The average of all nondiffusion-weighted volumes (referred to as the “b0 average” image) provided a complete anatomical image that was used to define masks and to register results onto a standard atlas. This image was used alongside the average of all diffusion-weighted volumes (the “b4K average” image) to define a whole-brain binary mask that excluded areas outside the brain and large fluid-filled ventricles and sulci. Thresholds were separately chosen and were applied to the b0 and b4K average images to remove brain tissue from the former and to remove fluid-filled spaces from the latter. This mask was created for each brain and was used to constrain tractography to follow tracts within the brain tissue. Masks were visually checked and were manually corrected. Image processing and analysis were performed using tools developed at the Centre for Functional Magnetic Resonance Imaging of the Brain (FMRIB Centre, University of Oxford) and were incorporated into FSL (www.fmrib.ox.ac.uk/fsl; Smith et al. 2004; Woolrich et al. 2009; Jenkinson et al. 2012).

### Defining Regions of Interest for Tractography

Using high-resolution postmortem b0 images of the dMRI scan, masks for region of interests (ROI) were manually delineated with reference to a standard atlas (Saleem and Logothetis 2012) and also to myelin-stained histological sections from the individual animals’ brain. The masks were drawn onto the b0 average images for each hemisphere using FSLView (v3.1). The same brains were scanned both in vivo and postmortem, but there can be changes in the brain shape after removal from the skull. Thus, in vivo FA images were registered to the corresponding postmortem FA images by using nonlinear registration (FSL, FNIRT; Andersson et al. 2010). The resulting nonlinear transform was then used to map postmortem ROI masks onto the in vivo dMRI space, followed by manual corrections, where required. Three kinds of masks were used to guide probabilistic tracking: Seed, Target, and Exclusion masks.

**Figure 1:**
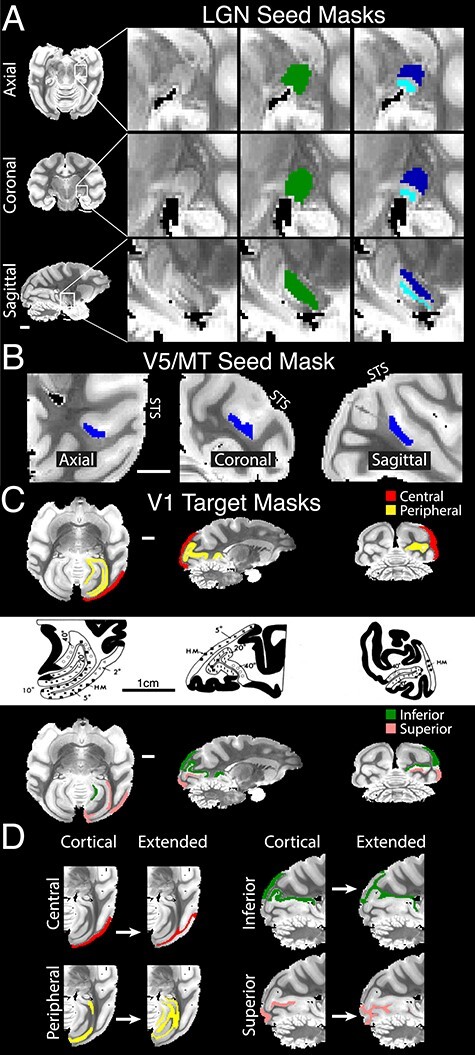
Tractography seed and target masks. (*A*) Axial (top row), coronal (middle), and sagittal (bottom) b0 image slices of the LGN (left column), with magnification (right columns). Seeds were defined using masks that covered either the whole of the LGN (green) or separately the magnocellular (light blue) and parvocellular (dark blue) layers. (*B*) Extrastriate visual cortical area V5/MT seed mask (blue). (*C*) Known retinal topography of V1 was used to define cortical target masks, either by eccentricity (central visual field, red; peripheral, yellow) or by elevation (inferior, green; superior, pink). The V1 topographic map from Van Essen et al. (1984) is shown for comparison (reprinted from Vision Research, 24, Van Essen, Newsome, Maunsell, The visual field representation in striate cortex of the macaque monkey: Asymmetries, anisotropies, and individual variability, page 444, Copyright (1984), with permission from Elsevier). (*D*) V1 cortical masks (“Cortical”) were used to define a target mask that comprised the immediately neighboring white matter (“Extended”). Well-connected voxels in white matter were identified using tractography by seeding the original V1 cortical masks and run tractography over a very short distance only (see Materials and Methods). Thus, “extended” masks occupy voxels directly underneath the cortical target in the white matter, which would have to be crossed on approach to the specific V1 target. White and black scale bars are 1 cm. All MR images are from postmortem scans.

**“**Seed masks**”** were created for the LGN, including separate masks for the magnocellular and parvocellular subcomponents, and for visual area V5/MT, located in the STS of the macaque (Fig. 1*A*,*B*). The left hemisphere of each brain was processed histologically and was stained for myelin. Therefore, we were able to hand-draw a custom V5/MT mask for individual brains in the left hemisphere that matched the area of myelin-identified V5/MT. This accounted for intersubject variation in the size and precise location of V5/MT (Van Essen et al. 1981; Large et al. 2016). Nonlinear registration (FSL, FNIRT) was used to align a mirrored copy of the whole brain onto the original, brain image. Using this transformation, a V5/MT mask for the right hemisphere of each individual brain was prepared.

“Target masks” were defined using anatomical landmarks in each hemisphere to describe subregions of the primary visual cortex (V1) that served different portions of the visual field based on the functional maps from Van Essen et al.’s (1984) study. The first pair of masks was based on visual field eccentricity, with one mask corresponding to the central portions of the visual field (Central V1: eccentricities 0–11°) and the other corresponding to the peripheral portions of the visual field (Peripheral V1: eccentricities ≥12°) (Fig. 1*C*). The second pair of masks was based on visual field elevation, with one mask corresponding to the lower visual field (inferior) and the other corresponding to the upper visual field (superior).

“Exclusion masks” were used to stop streamlines from running through the sulci, ventricles, or the contralateral hemispheres.

“Extended target masks”: Previous studies have indicated a path-length-dependent bias of probabilistic tractography (Liptrot et al. 2014). Our data showed a negative correlation between the distance of a voxel from the seed (along the streamline pathway) and the number of streamlines that passed through the voxel (average Spearman’s correlation coefficient = −0.79, SD = 0.15; postmortem, 12 hemispheres, 8 seeds/target combinations, *n* = 96; all coefficients *P* < 0.02). Accordingly, we implemented a simplified version of the ICE-T algorithm by Liptrot et al. (2014) to define extended target masks. This procedure defines masks that include white matter voxels that are highly connected and close to the V1 cortical gray matter.

The connection strength of these white matter voxels was determined by seeding the original gray matter V1 masks (5000 samples per seed voxel, maximal curvature = ±90°, maximum number of steps = 24, and step length = 0.5 mm). No target was provided, but the usual exclusion mask was still used. White matter voxels with more than the median number of streamlines were incorporated into a new extended V1 target mask. Figure 1*D* shows separately the original targets in the V1 gray matter and the extension of these into the neighboring white matter. Using an extended target mask amplified the number of streamlines reaching V1 without loss in accuracy for mapping topography (see Supplementary Table S1). The use of extended target masks, in particular, facilitated streamlines to reach central visual field representations of V1 by bridging the narrow and sharply turning white matter tracts in rhesus macaques. Extended target masks also bridge regions of weaker diffusion direction in the white matter directly underneath cortical ROIs (mean FA: V5/MT = 0.40 [SD = 0.11]; central V1 = 0.47 [0.10]; peripheral V1 = 0.57 [0.10]; superior V1 = 0.51 [0.10]; inferior V1 = 0.52 [0.10]) in comparison with a pure tract of white matter (corpus callosum FA = 0.80 [0.01]).

### Probabilistic Tractography for In Vivo and Postmortem dMRI Datasets

We performed probabilistic tractography on the diffusion-weighted images in the native space of each individual brain using the implementation in FSL. This uses the Ball&Stick model for voxel-wise fitting of fiber orientation distributions (FOD) (BedpostX function, model 1, Behrens, Woolrich, et al. 2003; Behrens et al. 2007). Probabilistic tractography was performed by sampling the fitted FODs (Probtrackx2 function; Behrens, Woolrich, et al. 2003; Behrens et al. 2007) to generate virtual processes, called streamlines which propagate from voxels in a seed region, through white matter pathways, to a target region. For an analysis of the anisotropic volume fraction contributed by primary and secondary fibers for the in vivo LGN to V1 tracts, see Supplementary Figure S1.

To determine an appropriate set of tractography parameters, we systematically evaluated outcome for the predicted maps of LGN visual eccentricity (see below) for a single postmortem hemisphere (M130, left hemisphere). The number of steps was varied in relation to the step length to limit the maximum permitted streamline length as a percentage of the total anterior-to-posterior brain length (~70–75 mm). The following parameters were used:

Streamline length: 50%, 75%, 100%, or 125% (of total brain length).

Step length: 0.0625, 0.125, 0.25, or 0.5.

Curvature threshold: 0.0, 0.2, 0.4, or 0.6.

As described below, the predicted maps of visual eccentricity were validated against the neurophysiological LGN atlas (Erwin et al. 1999) for each parameter combination. This process was carried out for tractography sessions using cortical gray matter targets only or using extended target masks (Fig. 1 and below) and for before or after probability density normalization (pre-/post-PDF) (see below). Our main tractography parameters are based on this analysis (see Supplementary Fig. S2).

Therefore, in the analysis for this paper, Probtrackx2 was executed using modified Euler integration, a maximal curvature threshold of ±90° (i.e., cosine of 0), and a maximum number of steps equivalent to 75% of the brain’s anterior–posterior length (typically around 110 steps, i.e., 55 mm). The choice of curvature threshold was to compensate for the large step size, while a maximum length was imposed to prevent streamlines from visiting many other areas before reaching the target and to prevent folding.

For postmortem tractography, 3 × 10^5^ streamlines were generated per seed voxel with 0.5 mm step length. For in vivo tractography, 2.4 × 10^6^ streamlines were generated per seed voxel with 1 mm step length; the increased sample count maintained the same density of streamlines per unit volume of tissue for the in vivo tractography, which had larger voxels (1 mm^3^ in vivo vs. 0.125 mm^3^ postmortem). FSL settings for all other parameters are summarized in Supplementary Table S2. We did not use distance correction in FSL but used extended target masks and probability density normalization (see below).

As a control to check the specificity of the tractography data, we removed in one animal (M130 postmortem) the contralateral exclusion mask and tracked from the LGN to the contralateral central and peripheral V1 extended target masks. Of more than 4.50 × 10^8^ streamlines, only four reached the contralateral central or peripheral V1 cortex.

### Probability Density Function for Segmenting Seed Areas

**Figure 2:**
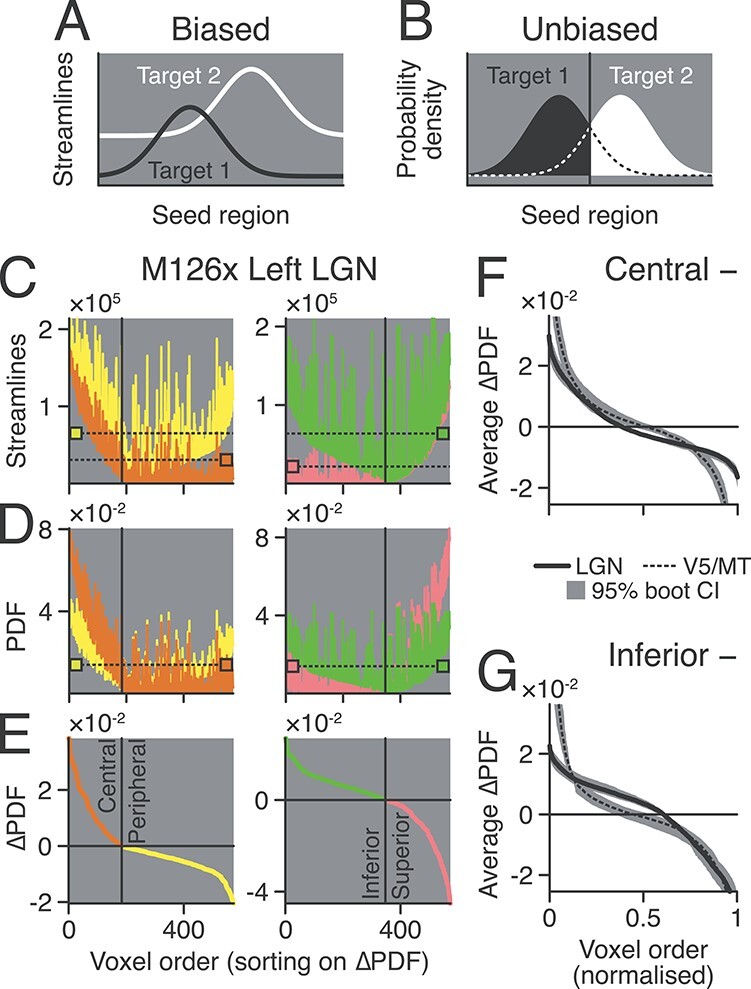
Probability Density Function (PDF) (*A*) Illustrating potential bias of probabilistic tractography. A streamline frequency map from a seed region (position mapped on *x*-axis) to Target 1 (black) might overall show fewer streamlines than to Target 2 (white); this could occur if Target 2 was closer to the seed. (*B*) The effect of normalizing streamline counts by estimating the PDF. Each part of the seed is assigned a visual-field representation (black/white area) based on which target it received the highest probability density from either Target 1 (black area) or Target 2 (white area). (*C*) Example streamline frequency maps from one hemisphere (left LGN of M126, postmortem) when targeting either representations of central (orange), peripheral (yellow), inferior (green), or superior (pink) visual field in V1. Colored squares with dashed lines show the average streamline frequency for each map. In (*C*–*E*), seed voxels were ordered the same way on the *x*-axis by the difference of PDF values in (*E*). (*D*) Same as (*C*) but showing the PDF estimates. Notice that the averages (squares/dashed lines) are now at the same level. (*E*) The difference of PDF maps in (*D*) showing the central minus peripheral PDFs (left panel) and inferior minus superior PDFs (right panel). Colors indicate which visual field representation the seed voxels were assigned to. (*F*) The population average difference of PDFs for visual eccentricity maps in LGN (solid) and V5/MT (dashed). (*G*) Same as (*F*), except showing the average PDF difference for visual elevation maps. Gray shading in (*F*) and (*G*) shows 95% bootstrap CIs.

The probabilistic algorithm produced a streamline frequency map of the seed area, showing how many streamlines reached the targets from each voxel. V1 targets contained different visual field representations, so labeling each seed voxel according to the target toward which it sent out the greatest frequency of streamlines provides a prediction of the visual topography of the seed area. Rather than using raw counts of streamlines, we calculated probability density functions (PDFs) to normalize the data. When a seed region is used in two sessions of probabilistic tractography with two different targets, one of these targets may receive a higher absolute number of streamlines simply because one target is larger than the other or because it is closer to the seed (Liptrot et al. 2014). Thus, labeling seed voxels based on the raw number of streamlines is likely to bias estimates of which parts of the seed region are better connected to the target against targets with a lower baseline level of hits. We normalized the streamline frequency maps by applying moderate spatial smoothing (3D Gaussian convolution kernel, 0.5 mm SD postmortem, and 1 mm SD in vivo) to overcome regions of sparse sampling and then computed the probability density of each seed voxel:

(1) \begin{equation*} {p}_i={s}_i\, /\left(\ S\times v\ \right)\ \mathrm{and}\ S=\sum{s}_i, \end{equation*}

where *s_i_* is the number of streamlines from the *i*th seed voxel that reached a designated target, *S* is the total number of streamlines from all seed voxels that reached the same target, *v* is the volume of a voxel (0.125 mm^3^ postmortem, 1 mm^3^ in vivo), and *p_i_* is the probability density of the *i*th voxel. Figure 2 illustrates how normalized streamline counts are expected to produce PDF estimates with similar baselines and peaks for connections of similar strength. For this analysis, a PDF map was computed for each seed and target combination. Seed voxels were then labeled according the target that assigned the highest probability density.

Figure 2*C*–*E* shows the normalization of an example seed area (LGN, left hemisphere, M126 postmortem) when targeting either central (orange) or peripheral (yellow) V1. The streamline count was biased in favor of peripheral V1, which had a higher baseline average than central V1. After estimating the PDF from each set of streamline counts, the baseline level was normalized, but the shape of each curve stayed the same. By subtracting the peripheral from central PDFs (Fig. 2*E*, ΔPDF), we can visualize how each seed voxel was labeled. The same technique was used to predict the visual field representation of every seed voxel for each pair of V1 targets; once to determine its visual eccentricity (V1 central vs. peripheral) and again to determine its visual elevation (V1 inferior vs. superior).

To summarize seed voxel labeling across all of our postmortem data, the population average difference of PDFs was found (Fig. 2*F*,*G*) by normalizing the voxel order number to between 0 and 1 and then using cubic-spline interpolation to correct for different seed sizes. For both the central-to-peripheral and inferior-to-superior visual field maps, there were consistently large parts of both the LGN (solid) and V5/MT (dashed) seed regions with a clear preference for one V1 target or the other (95% bootstrap confidence intervals [CIs], gray shading).

### Evaluating the Topographic LGN Predictions

The predicted maps of visual eccentricity and elevation in LGN were evaluated against a high-resolution (25 × 25 × 25 μm isotropic voxels) neurophysiological atlas of a rhesus macaque LGN (Erwin et al. 1999). The atlas was mapped by a combination of histological and electrophysiological techniques. We divided the atlas into two pairs of distinct regions based on visual field position. For one pair, we identified the parts of the atlas with either central (0–11°) or peripheral (≥12°) visual field eccentricity. For the other, we found the parts with visual field elevation that was either above (superior) or below (inferior) the horizontal meridian. This provided a benchmark against which the tractography-based maps of LGN could be directly compared.

First, each LGN seed mask was registered to a binary mask of the atlas (FSL, FLIRT, linear affine transformations); the same transformations were then used to register the predicted topographic maps onto the atlas. Parts of the registered LGN that fell outside of the atlas were removed before further analysis. On average, 87.0% (SD = 4.61%) of the volume of registered LGN was retained. Finally, the percentage of correctly classified voxels was calculated for each identified subregion of the atlas (central, peripheral, inferior, and superior). This was calculated as the fraction of voxels labeled the same (e.g., central) in the predicted map and the neurophysiological atlas over the total number of voxels in the neurophysiological atlas falling into this part of the visual field (e.g., central).

### Randomized Nonparametric Tests for Spatial Map Arrangements

We employed randomized, nonparametric resampling procedures (e.g., Efron and Tibshirani 1993; Nichols and Holmes 2002) to compare the spatial arrangement of the topographic maps. A random permutation test was used to estimate the significance of the voxel-wise similarity between pairs of topographic maps for postmortem dMRI versus LGN atlas. We calculated the percentage of voxels assigned to the same visual field representation in both maps. For each permutation, the topographic labels were randomly exchanged between voxels—separately for each map within the spatial confines of the ROI. Then, the percentage of voxels with the same topographic label in both maps was recalculated. This procedure was repeated 10 000 times, yielding a distribution of percentages from which we could estimate the CIs and *P* values. An advantage to this approach was that no assumption had to be made about the chance level of similarity, which could potentially depend on the balance of visual field representations in topographic maps.

We used the same approach to analyze the percentage overlap for the postmortem versus in vivo dMRI comparison.

### Connectivity Probability

To compare the relative number of connections between a V1 target and the magnocellular or parvocellular layers of LGN, we calculated the probability that two areas were anatomically connected by computing the fraction of generated streamlines that reached the target:

(2) \begin{equation*} P=S\,/\left(k\times V\right). \end{equation*}

Here, *P* is the connectivity probability, *S* refers to the total number of streamlines from the seed that reach the target, *V* is the volume of the seed mask (in voxels), while *k* is the number of streamlines generated by each seed voxel.

### Classification Overlap Ratio

The classification overlap ratio (*R*_co_) compares the topographic maps predicted from dMRI and tractography between the in vivo dMRI data (*A*_in_) and the postmortem dMRI data (*A*_post_), calculated for each topographic label separately:

(3) \begin{equation*} {R}_{\mathrm{co}}={P}_{\mathrm{emp}}/{P}_{\mathrm{chance}}. \end{equation*}

Here, *P*_emp_ is the empirically measured proportion of seed voxels that are labeled in both *A*_in_ and *A*_post_ with the same topographic label (e.g., “superior”). *P*_chance_ is the probability that a seed voxel will be contained in *A*_in_ and *A*_post_, by chance:

(4) \begin{equation*} {P}_{\mathrm{chance}}={P}_{\mathrm{in}}{P}_{\mathrm{post}}+\left(\vphantom{{P}_{\mathrm{post}}} 1-{P}_{\mathrm{in}}\right)\ \left(1-{P}_{\mathrm{post}}\right). \end{equation*}

Here, *P*_in_ and *P*_post_ are the proportions of the seed mask that are occupied by either area (*A*_in_ or *A*_post_) alone.

### Standard Space

While all tractography was carried out in native space, for spatial normalization and presentation of the results in stereotactic alignment, results were transformed onto the 112RM-SL atlas of McLaren et al. (2009). Each postmortem b0 average image was registered onto the atlas (FSL, FLIRT, affine linear registration), and the resulting transformations were used to register the predicted topographic and PDF maps and related analyses. For 3D visualization of streamline density through the white matter, we obtained the transformation that maps the b0 average image for each hemisphere to a standard macaque brain (McConnell Brain Imaging Centre, Montreal Neurological Institute, McGill University; FSL, FNIRT), and the resulting transformation was used to register whole-brain streamline density maps to the Montreal Neurological Institute (MNI) standard brain (Frey et al. 2011). However, the comparison between postmortem and in vivo topographic maps was done in the postmortem b0 space of each individual hemisphere.

## Results

### Mapping Visual Topography in the LGN with Tractography

We tested whether we could correctly predict the retinotopic organization of the LGN, the main thalamic relay between the retina and the visual cortex, based on the specificity of connections with different parts of V1. Six macaque brains underwent high-resolution postmortem dMRI (voxel size 0.5 × 0.5 × 0.5 mm; Dyrby et al. 2011). Probabilistic tractography was conducted separately for each hemisphere with seeds set in the relevant LGN (Fig. 1*A*). Tractography based on dMRI binds together a sequence of local diffusion vectors from dMRI into spatially extended streamlines, which are candidate axonal projections.

We examined representations of elevation and eccentricity in the contralateral visual field. For elevation, streamlines targeted either the superior or inferior visual field quadrant of the ipsilateral V1 (Fig. 1*C*,*D*); for eccentricity, target ROI covered either the central 11° of visual field or eccentricities >12° for the peripheral visual field (Fig. 1*C*,*D*). The probability density of streamlines hitting one of the pair of ROIs was computed, and the visual mapping of each LGN voxel was assigned accordingly.

**Figure 3:**
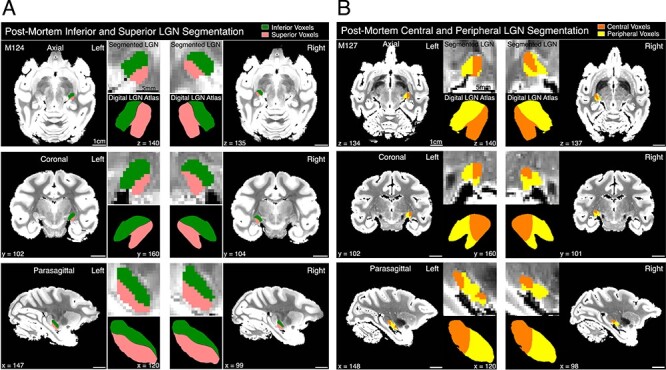
LGN topography. (*A*) The predicted elevation map for the LGN in brain M124. Full brain and magnified views give the midpoint slices of the LGN in each plane (rows) when compared with corresponding midpoint slices the LGN atlas (Erwin et al. 1999). Colors show the visual field representation in each map (inferior visual field, green; superior, pink). White scale bars are 1 cm, black scale bars (top left LGN images) are 5 mm. (*B*) The predicted eccentricity map for LGN in brain M127. The same as in (*A*), except that orange maps the central 11° of visual field and yellow maps peripheral visual field.

**Figure 4:**
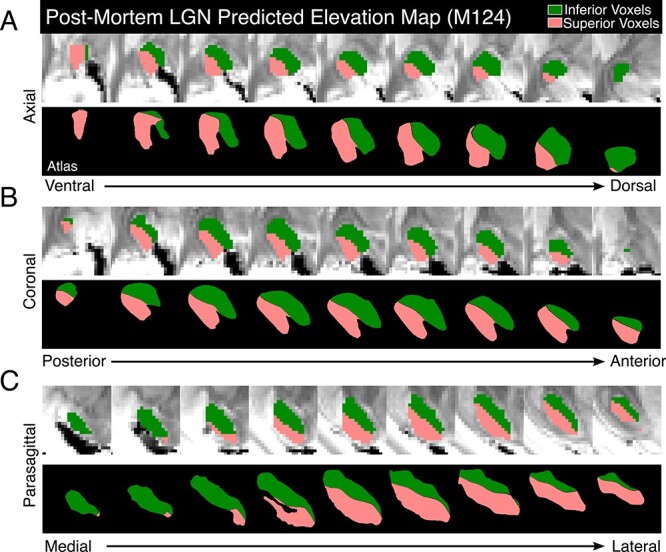
Slice-by-slice examination of LGN elevation map. The predicted map (M124, right hemisphere) is shown above corresponding sections in the LGN atlas (Erwin et al. 1999) in the axial (*A*), coronal (*B*), and sagittal (*C*) planes of view. Colors are as in Figure 3*A*.

**Figure 5:**
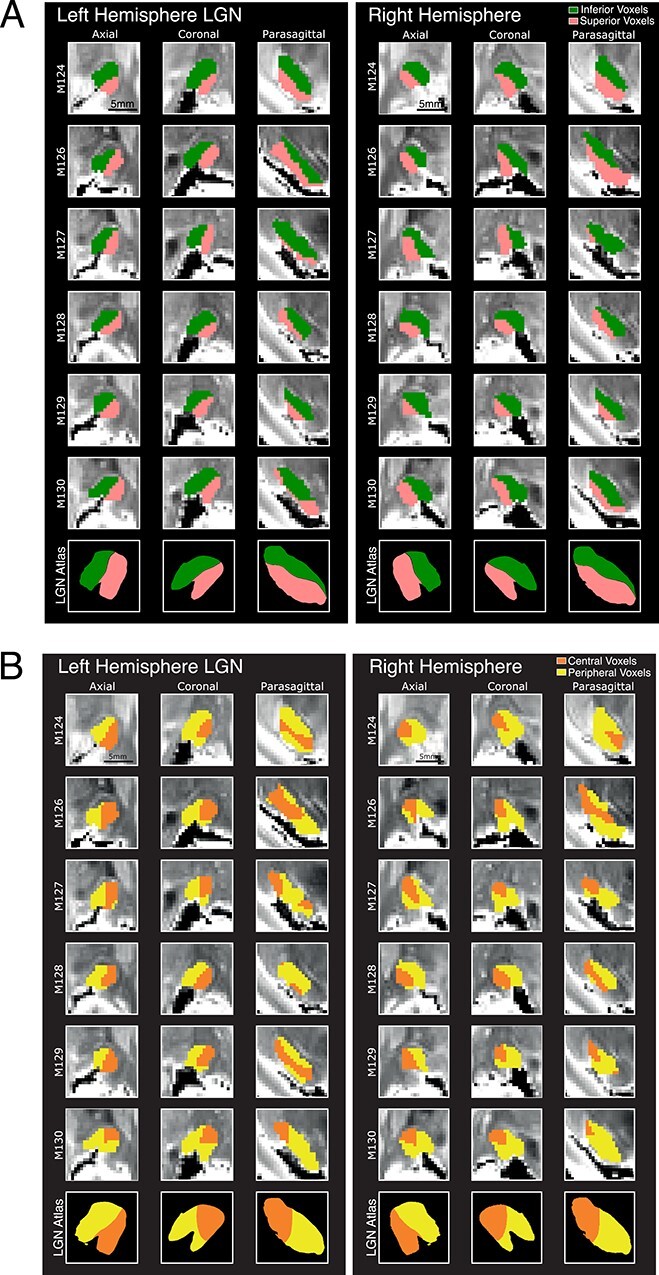
LGN Topographic maps for all 12 hemispheres. (*A*) Predicted LGN elevation maps. Middle slices of LGN are shown for each brain (rows), all three planes of view (columns), and each hemisphere (panel). The bottom row shows corresponding points in an LGN neurophysiological reference atlas (Erwin et al. 1999). Color scheme is the same as in Figures 3*A* and 4. (*B*) Predicted LGN eccentricity maps. The same layout as (*A*), but colors are as in Figure 3*B*.

Elevation maps based on the comparison of these probability densities of streamlines divided the LGN into superior and inferior visual field quadrants in accordance with previous neurophysiological data published as a functional LGN atlas (Erwin et al. 1999). Figure 3*A* shows an example (M124) in which the inferior visual field representation is mapped onto the dorsal-medial half of LGN (green) and the superior visual field is mapped onto the ventral-lateral half (pink). The border occupied a central position, where the horizontal meridian would be expected according to the functional LGN atlas (Erwin et al. 1999). A slice-by-slice examination for the elevation map (Fig. 4) shows that the pattern of our topographical prediction matched the atlas throughout the LGN. In particular, there was a smooth transition from the middle of LGN to the edges. The LGN topographic map for elevation was consistent in all 3 stereotactic planes across all 12 hemispheres we examined (Fig. 5*A*).

Next, we determined the visual eccentricity map of LGN using the same analysis. We targeted either the central or peripheral portions of V1 in tractography sessions that were seeded in the LGN. Again, the predicted maps comprised two distinct regions: the central visual field (orange) was mapped onto the lateral-posterior portion of LGN, and the peripheral visual field eccentricities (>12°) mapped onto the medial-anterior portion of the LGN (yellow) (Fig. 3*B*, for one example brain, M127). As for the elevation maps, the relative positions of these visual field divisions established through dMRI and tractography generally agreed with the functional LGN atlas (Erwin et al. 1999) for most hemispheres very well (*n* = 9/12; Fig. 5*B*) (see also Supplementary Fig. S3 for overlaid example sections). However, in some hemispheres (e.g., M124 left), tractography predicted central representation where peripheral representation was expected, and this was particularly evident when viewed in the parasagittal plane.

### Accuracy of Maps

**Figure 6:**
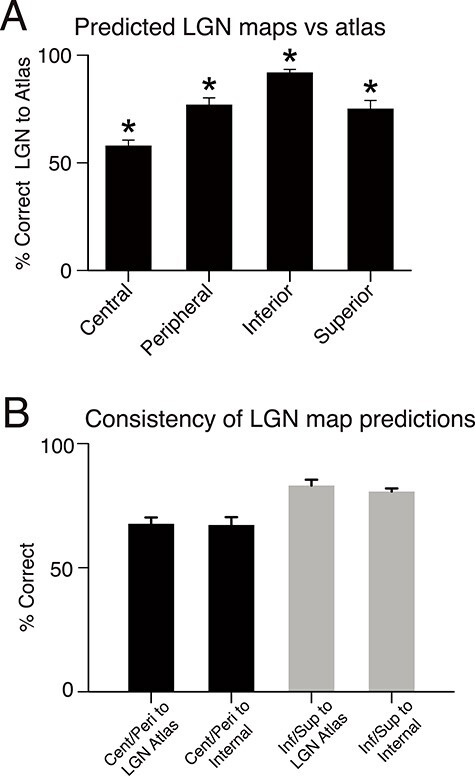
Quantifying LGN prediction accuracy based on dMRI. (*A*) The average percentage of a neurophysiological atlas of the LGN (Erwin et al. 1999) that was matched by the topographic map predictions based on dMRI and tractography. This comparison was done separately for each visual field division of the atlas: central visual field, peripheral, inferior, and superior. Central (mean = 58.1% [SD = ±8.8%], one sample *t*-test, *P* = 0.008), peripheral (mean = 77.1% [±10.7%], *P* < 0.001), inferior (mean = 92.0% [±4.7%], *P* < 0.001), and superior (mean = 75.2% [±13.0%], *P* < 0.001) voxels of the LGN were mapped to the correct part of the visual field map significantly above chance (^*^t-test, *P* < 0.01). (*B*) Percentage of LGN map predictions that was correctly labeled according to the neurophysiological atlas (left bar in each colored pair; based on data in (*A*)) are compared to internal consistency of predicted maps derived with an internal LOOCV approach (right bar in each pair). Error bars show standard error of the mean.

We quantified the accuracy of the topographic maps predicted from dMRI-based tractography by first registering them onto the 3D functional atlas of the LGN (Erwin et al. 1999) and then by making a voxel-wise comparison. We examined each visual field division separately (central, peripheral, inferior, and superior) to establish the percentage of voxels that were correctly assigned (%correct; Fig. 6*A*). On average, maps predicted by the streamline probability densities agreed well with the functional LGN atlas (mean correct percentage of volume = 75.3% [SD = ±11.5%]). The percentage of correctly assigned voxels was above chance for each subdivision of the visual field (Fig. 6*A*) (one-way *t*-test, *P* < 0.001). Predicted elevation maps were more accurate (mean %correct = 83.1% [±8.2%]) than eccentricity maps (mean %correct = 67.7% [±13.7%]; paired *t*-test, *P* < 0.001). Dice coefficients yielded comparable results (Supplementary Fig. S4).

When we tested whether the predicted topographic maps based on tractography contained appropriate proportions for different visual field representations, we found for maps based on tractography that, on average, 38.3% (SD = ±4.5%) of LGN mapped to the central 11° of visual field and 61.7% (±4.5%) to the peripheral visual field (>12°); 40.5% (±5.6%) superior and 59.5% (±5.5%) inferior. By contrast, in the neurophysiological LGN atlas (Erwin et al. 1999), 48.4% of LGN was representing the central 11° and 51.0% the peripheral visual field; 50.5% was representing superior and 48.6% inferior visual field. Therefore, the tractography map predictions underestimated the LGN representation of the central portion of the eccentricity map and the superior field portion of the elevation map. Even so, when we checked the accuracy of individual topographic maps (central–peripheral; superior–inferior) to the Erwin atlas with a random permutation test, 23 of 24 topographic maps were significantly better than chance (*P* < 0.05) with a rejection for only one map for one hemisphere (M128, left hemisphere, eccentricity map, 49.2%).

These results show that tractography can be used to determine the correct topographic organization of LGN for elevation and eccentricity based on how different parts of the visual field representation in V1 can be connected to the LGN by tractography. Our results were quite robust across different methodological choices, like overall streamline length and whether target masks extended into white matter (see Materials and Methods and Supplementary Table S1). We used PDF normalization for all of these analyses.

To determine the internal consistency of the predicted LGN topographic maps, a Leave-One-Out-Cross-Validation (LOOCV) analysis was carried out in which the topographic map from each hemisphere (the test set) was compared with a composite average of the predicted maps from all other hemispheres (the model set), which was registered to the same native space as the test hemisphere. The percentage of voxels that were assigned the same visual field label between the test hemisphere and the model set was computed as described previously for the topographic maps versus the LGN atlas. The mean similarity between the test set and the model set was computed separately for each hemisphere and the central/peripheral and inferior/superior map segmentations. The results demonstrate a good level of internal consistency (Fig. 6*B*)—comparable to the similarity of the topographic maps with the LGN neurophysiological atlas by Erwin et al. (1999) (Fig. 6*A*) (two-way ANOVA; comparison type effect, *F*(1, 11) = 0.37, *P* = 0.56; topographic map type effect, *F*(1, 11) = 55.33, *P* < 0.001; interaction, *F*(1, 11) = 0.32, *P* = 0.59). The less consistent results that we found for the central/peripheral segmentation rather than the inferior/superior are consistent with the lower accuracy achieved for the predicted central/peripheral topographic maps.

### Geniculo-Cortical Connections: Mapping Magno- and Parvocellular Connections between LGN and V1

We further tested whether tractography would show differences in the distribution of streamlines originating from the magnocellular or parvocellular layers of the LGN and targeting either the central or peripheral visual field representation of V1. The estimated connectivity probability to either V1 target was much stronger when seeding parvocellular LGN (mean = 0.344 [SD = 0.174]) as compared with magnocellular LGN (mean = 0.014 [SD = 0.024]), which is in general agreement with known differences in afferent numbers (Le Gros Clark 1941; Connolly and Van Essen 1984; Malpeli et al. 1996).

**Figure 7:**
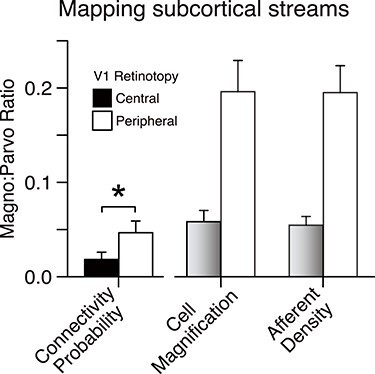
Mapping magnocellular and parvocellular LGN–V1 connectivity. We used our dMRI data and tractography analysis to estimate the magnocellular/parvocellular ratio in input to different V1 targets. This graph shows on the left the connectivity probability for streamlines derived from tractography seeded in the LGN in magnocellular over parvocellular layers to different parts of the visual field in V1 (central visual field, black; peripheral, white). The previously established magno−/parvocellular ratio for cell magnification factor and of cortical afferent density in central (gray) and peripheral visual V1 (white) are shown on the right for comparison. Magnification factor and afferent density were taken from Malpeli et al. 1996 (their fig. 10 and 12). ^*^significantly different (paired *t*-test, *P* = 0.017). All error bars show standard error of the mean.

Furthermore, our measure of connectivity probability showed a similar magnocellular/parvocellular (M/P) ratio as reported by others previously for afferent density and magnification factor (average M/P ratio: central V1 target = 0.018 [SD = 0.03]; peripheral V1 = 0.047 [0.04]; paired *t*-test, *P* < 0.001; Fig. 7). Our data also suggest that a larger proportion of magnocellular afferents connect to peripheral V1. Thus, probabilistic tractography appears to reflect the distinct structural connectivity profiles of two functional subdivisions of the geniculo-cortical visual pathway.

### Geniculo-Cortical Connections: Topographic Organization Maintained throughout the Streamline Bundles

**Figure 8:**
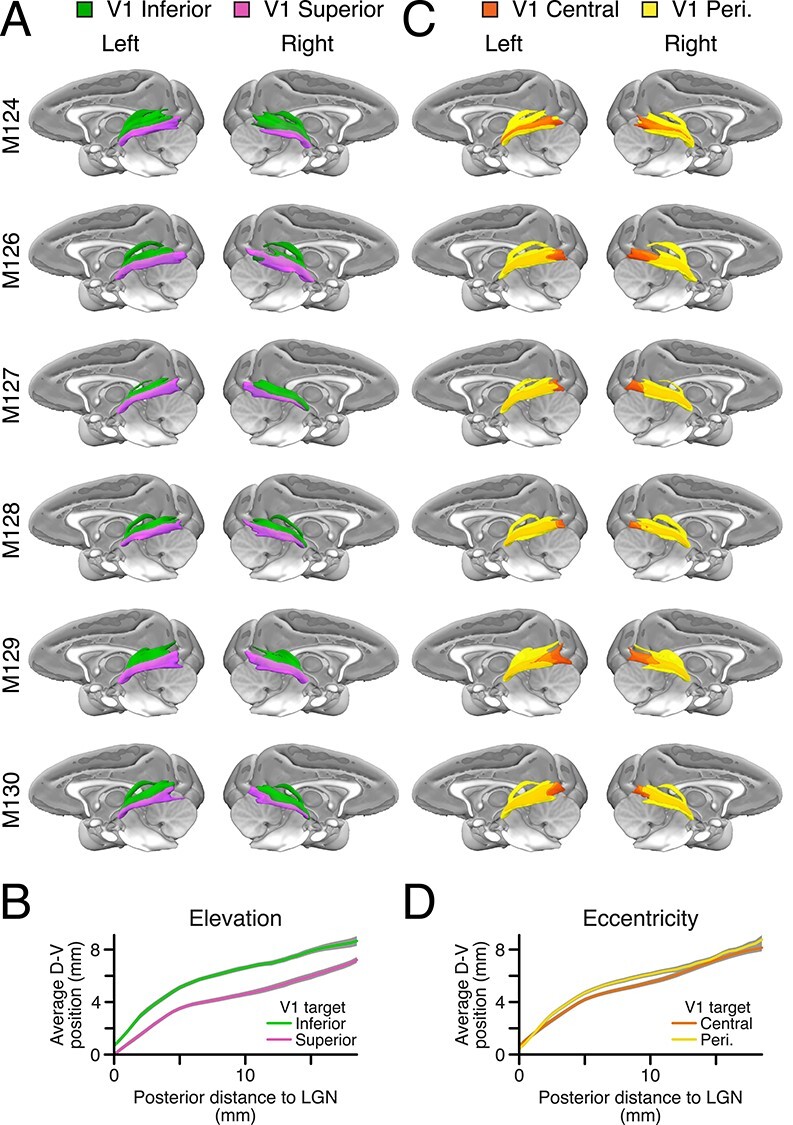
Streamline density maintains topographic organization. (*A*) Density of two sets of streamlines that were seeded in LGN and targeted either V1 inferior (green) or superior visual field (purple) (only voxels with density values in the top 90–100 percentile are shown). (*B*) Population average of the weighted average dorsal-ventral position of the streamlines in (*A*). (*C*) Same as (*A*) but targeting V1 central (orange) or peripheral (yellow). (D) Same as (*B*) but using the streamlines in (*C*). In (*A* and *C*), 3D surfaces show the volume containing voxels with the top 10% of streamline densities, created using the MR Comparative Anatomy Toolbox (Mr Cat) (Donders Institute and University of Oxford, UK; Mars et al. 2015) and the MNI macaque brain atlas (McConnell Brain Imaging Centre, McGill University; Frey et al. 2011).

We visualized the main trajectories that streamlines took from LGN to V1 by counting the number of streamlines passing through each voxel. Streamline density maps for each hemisphere and V1 target were normalized by the respective map’s peak value. All maps were registered to MNI standard space (McConnell Brain Imaging Centre, McGill University; Frey et al. 2011). The voxels with density values in the top 90–100 percentile are shown in Figure 8*A* and *C*.

We plotted the average of the dorsal-ventral position of the streamline densities, shown in Figure 8*A*, for each coronal slice. Population averages of streamline densities obtained from probabilistic tractography maintained a distinct topological arrangement along the entire trajectory from the LGN to V1 (Fig. 8*B*). Streamlines originating from ventral LGN (Fig. 8*A*, purple), representing the superior visual field quadrant V1, remained ventral to the streamlines from dorsal LGN (Fig. 8*A*, green) representing inferior visual field all the way to their V1 target (mean difference = 1.68 mm [SD = 0.73 mm], paired *t*-test, *P* < 0.001). The dorso-ventral patterning of streamlines connecting to central and peripheral visual field representations in V1 was also consistent from hemisphere to hemisphere. While the streamlines targeting central V1 (Fig. 8*C*, orange) appeared to be enveloped by those targeting peripheral V1 (Fig. 8*C*, yellow), the population average streamline density terminating in the peripheral visual field representation in V1 was slightly more dorsal (mean difference = 0.41 mm [0.50 mm], paired *t*-test, *P* < 0.003; Fig. 8*D*). These results indicate that tractography in postmortem brains can reveal aspects of the visual topography within these white matter tracts.

### Cortico-Cortical Projections: Predicting V5/MT Visual Topography from Postmortem dMRI and Probabilistic Tractography

**Figure 9:**
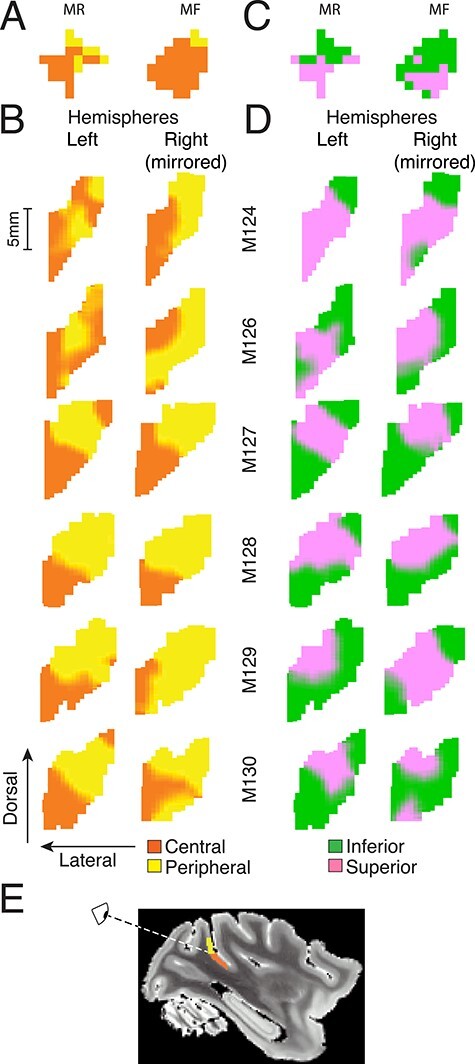
V5/MT topography. (*A*) Average RF eccentricity of isolated V5/MT neurons recorded extracellularly using single electrodes in the left hemisphere of behaving monkeys (MR and MF; Krug et al*.* 2004). Squares show the average from different grid positions (1 mm spacing) in the recording chamber, which was angled at 20°. RFs were either in the central (orange) or peripheral (yellow) visual field. (*B*) The predicted V5/MT eccentricity maps based on postmortem dMRI. Maps were projected at 20° to match the electrophysiology data. (*C*) Same as (*A*) but showing whether RFs were in the inferior (green) or superior (purple) visual field. (*D*) Same as (*B*) but showing the predicted V5/MT elevation maps. (*E*) Illustration of view onto V5/MT topographic map shown in (*A*–*D*).

To test whether we can extend these techniques to cortico-cortical connections that are known to be sparser, we evaluated the quality of predictions of the visual topography of extrastriate cortical area V5/MT based on its connections to different parts of the V1 topographic map. The same mapping technique was used as for LGN, except that streamlines were seeded in extrastriate visual area V5/MT. The borders of cortical area V5/MT were defined for each brain with reference to Gallyas-stained histological slices revealing the pattern of myelination, which were taken from the left hemisphere of the brain. This was done to address intersubject variation of cortical location and size of V5/MT (Van Essen et al. 1981; Large et al. 2016). The predicted topographic maps based on tractography were compared with neurophysiological receptive field (RF) maps obtained in previous experiments (Krug et al. 2004) and analyzed in Parker et al. (2016). In those earlier experiments, electrode penetrations took a posterior approach to V5/MT through a recording chamber that was angled at 20**°** above the horizontal plane, which was also the electrodes’ trajectory. The perspective of the functional map (Fig. 9*A*,*C*) is based on a viewpoint (Fig. 9*E*) looking down into the recording chamber toward a slightly tilted coronal view of V5/MT from behind the monkey’s head (see also Parker et al. 2016). For comparison, topographic maps predicted from tractography to distinct visual field representations in V1 were also projected onto a 2D plane that was tilted by 20° as if viewed from the same posterior vantage point.

Figure 9*A* shows the average eccentricity of V5/MT RFs along each electrode penetration site for two different animals. The electrophysiological RF maps show that the central visual field was represented in the ventral-lateral part of V5/MT—while the periphery had a dorsal-medial representation (see also Van Essen et al. 1981; Kolster et al. 2009). Almost all topographic maps predicted from tractography show the same basic organization, with the central visual field region positioned ventro-laterally and the peripheral visual field dorso-medially (Fig. 9*B*).

In the neurophysiologically recorded RF maps for elevation (Fig. 9*C*), ventral V5/MT represented the superior contralateral quadrant and dorsal V5/MT represented the inferior contralateral quadrant. The upper parts of our dMRI- and tractography-derived elevation maps (Fig. 9*D*) had a similar arrangement; the dorsal tips were mapped as inferior and superior was mapped just below that. However, ventral parts of V5/MT were often mapped to inferior visual field, too. This could potentially indicate a map reversal and therefore that we entered a neighboring cortical area. We cannot rule out the possibility that the myelin-defined borders of V5/MT might spill outside the true V5/MT to incorporate parts of neighboring areas (Glasser and van Essen 2011).

### Topographic Map Orientation for LGN and V5/MT

**Figure 10:**
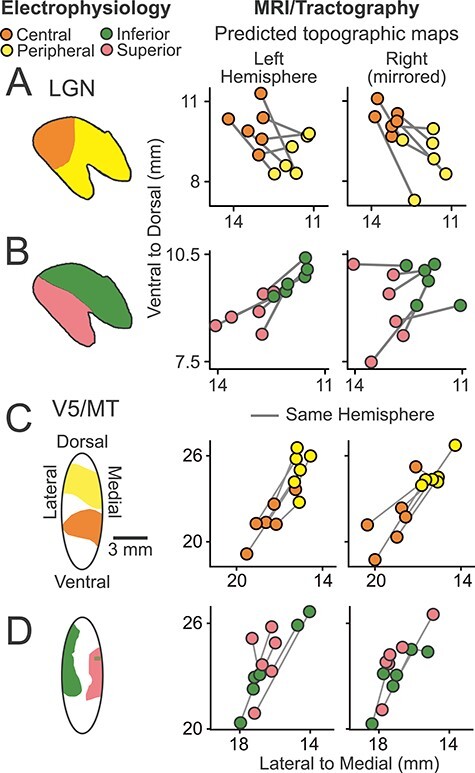
Topographic map orientations for LGN and V5/MT. (*A*) LGN topography maps of visual field eccentricity are shown in the coronal plane. In the right-hand panels, coronal projections are plotted at the average position (circles) of the central (orange) and peripheral (yellow) visual field representations obtained with dMRI and tractography. Data from the same hemisphere are linked (gray lines), indicating the main axis of the map. An electrophysiological LGN map is shown for comparison (left, LGN atlas, Erwin et al. 1999). (*B*) Like (*A*) but showing relative LGN elevation mapping for inferior (green) and superior (pink) visual field. (*C*) Conventions as in (*A*) but showing a V5/MT electrophysiological eccentricity map (left, redrawn from Van Essen et al. 1981 with permission) compared to the maps we obtained with dMRI and tractography (right). (*D*) Like (*C*) but showing the V5/MT elevation maps. Predicted topographic maps were all registered to the 112RM-SL atlas (McLaren et al. 2009) before taking average positions.

To further investigate the accuracy of the cortical and LGN topographic maps generated, we analyzed the relative orientation of the different parts of the visual field maps within these areas. We registered our tractography-derived maps onto the 112RM-SL atlas (McLaren et al. 2009) to allow comparison of data across hemispheres. Then, we calculated the average voxel position of different visual subfields (central, peripheral, inferior, and superior) (Fig. 10: shown in the coronal plane) for each binary topographic classification mask (e.g., Figs 3 and 9). When compared to the neurophysiological LGN atlas (Fig. 10*A*,*B*, left; Erwin et al. 1999) and the neurophysiological maps of V5/MT (Fig. 10*C*,*10D*, left, Van Essen et al. 1981; see also Fig. 9*A*,*C*), the intrinsic organization of our topographic maps (Fig. *10A–D*, right) was consistent from hemisphere to hemisphere and generally matched the known functional maps.

In the LGN, central visual field was dorso-laterally positioned compared to peripheral visual field, and the inferior visual field was dorso-medial relative to superior field as expected from the neurophysiological LGN atlas (Erwin et al. 1999) (Fig. 10*A*,*B*). The distance estimate from the classification masks (mean central vs. peripheral = 2.06 mm [SD = 0.79 mm]; inferior vs. superior = 1.56 mm [0.21 mm]) was also similar to the neurophysiological LGN atlas (Erwin et al. 1999) with a distance of 2.75 mm from central to peripheral and 2.25 mm from inferior to superior.

For V5/MT, our topographic predictions based on tractography placed central field consistently more ventral than peripheral field, and the distance estimates from the average voxel positions were comparable to previous observations (Van Essen et al. 1981, their fig. 12A) (Fig. 10*C*). But no consistent arrangement of the elevation maps was found (Fig. 10*D* right). Instead, our predicted maps indicated inferior visual field representations at dorsal and ventral perimeters of V5/MT in 10 out of 12 hemispheres (Fig. 9*D*). One potential source of this error might be a misalignment of the V5/MT seed mask in relation to map reversals in the V5/MT, FST, and MST clusters (Kolster et al. 2009). With the exception of the inconsistent mapping of visual field elevation in V5/MT, probabilistic tractography in postmortem brains produced topographic maps of the correct polarity for LGN and V5/MT when taken across the number of cases that we studied.

### Comparing Postmortem and In Vivo dMRI-Based Tractography

**Figure 11:**
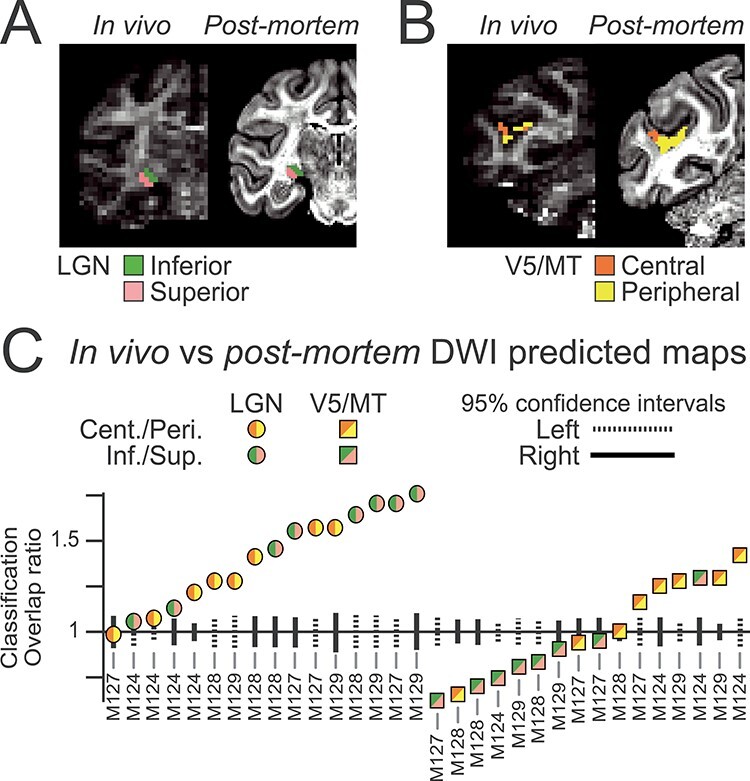
Comparing predictions of topographic maps based on in vivo and postmortem dMRI. Side-by-side comparison of the predicted LGN elevation (*A*) and V5/MT eccentricity (*B*) maps from an example brain (M129) scanned both in vivo (left) and postmortem (right), shown on top of coronal slices of the FA images. (*C*) Voxel-wise comparison of predicted eccentricity (orange/yellow) and elevation (pink/green) maps from in vivo and postmortem dMRI, for LGN (circles) and V5/MT (squares). Classification overlap ratio is 1 when the overlap of the two topographic map classifications could be achieved by chance. 95% CIs (lines; left hemisphere, dashed; right, solid) show the level of similarity expected by chance.

Obtaining dMRI in vivo is more challenging than postmortem acquisition because scan times are limited, the image resolution is usually lower, and the data contain physiological noise and motion artifacts. All of these factors result in images with lower signal. However, since dMRI provides one of the few methods to investigate in vivo connectivity, it is important to quantify the correspondence between postmortem and in vivo data. We therefore directly compared the topographic maps we obtained with postmortem dMRI for the LGN and V5/MT to in vivo dMRI data collected earlier from the same brains. As this is a within animal comparison, the two scans should result in very similar topographic maps based on connectivity to V1. For example, Figure 11*A* and *B* show clear similarity of the in vivo and postmortem LGN elevation (Fig. 11*A*) and V5/MT eccentricity (Fig. 11*B*) maps for one hemisphere (M129, right).

We analyzed in vivo data from four of the brains (eight hemispheres) for which we obtained postmortem data. Other than step length, which was matched to voxel size, the tractography analysis for in vivo dMRI was conducted using the same parameters as for postmortem dMRI. We registered in vivo tractography results to postmortem data using a nonlinear transform and nearest-neighbor interpolation to up-sample to 0.125 mm^3^, followed by probability density transformation and hard segmentation of topographic maps. This allowed a voxel-wise comparison (at 0.5 mm isotropic) of the in vivo and postmortem tractography-derived topographic maps from the same hemispheres. For each hemisphere, we determined the extent to which the empirically measured proportion of seed voxels that are labeled with the same topographic label (e.g., “superior”) (classification overlap ratio >1) and whether these results could have been attained by chance (CIs for classification overlap ratio = 1) (Fig. 11*C*).

When comparing the overlap of voxels labeled as belonging to the same part of the visual field representation, in vivo and postmortem maps were similar to each other overall, but the LGN maps showed much clearer correspondence (13/16) than the V5/MT maps (6/16). For V5/MT eccentricity, more maps showed significant correspondence between postmortem and in vivo dMRI (5/8) than for V5/MT elevation (1/8).

When compared to the postmortem topographic map classifications, we could delineate in vivo LGN topographies and V5/MT eccentricity maps much better than chance but not the V5/MT elevation maps. These results suggest that in vivo dMRI data may be sensitive enough to resolve within-area topography, in particular, for the geniculo-cortical projection, but is more challenging for some features of cortico-cortical projections. There are, however, features of this comparison that are less impressive, as in some cases for the comparisons of V5/MT overlap, there appeared to be some biases that resulted in a number comparisons of overlap that were apparently below chance predictions (overlap parameter <1). We assume that the bias lies in the in vivo measurements for V5/MT and note particularly that the majority of these cases arise from inferior–superior visual field comparisons.

## Discussion

Using probabilistic topography across a population of individual dMRI scans, we were able to make successful structural predictions of the topographic maps for visual field elevation in the LGN, for central versus peripheral fields of LGN and V5/MT, and the relative numbers of magno- and parvocellular connections from LGN to V1 at different eccentricities. We used “gold standard” topographic maps from electrophysiology to provide a baseline for these comparisons. The best results were obtained from high-resolution and high-quality postmortem dMRI datasets acquired at 4.7T, but key features of the predicted visual maps could also be demonstrated using lower resolution, shorter duration in vivo dMRI with a clinical 3T MRI scanner. We found that, in principle, both postmortem and in vivo dMRI in combination with probabilistic topography are sensitive enough to resolve intra-area topographic maps based on connectivity. However, in individual cases the predicted maps appear to be inconsistent or incorrect.

One question that motivated this study was whether the performance of dMRI and tractography (Basser et al*.* 2000; Kier et al*.* 2004; Jones 2010; Jones et al. 2013; Thomas et al. 2014; Knösche et al. 2015, Maier-Hein et al. 2017) could be improved by using high-resolution, postmortem dMRI data (Dyrby et al. 2011; Ambrosen et al. 2020) and by optimization of the parameters for probabilistic tractography (Behrens, Woolrich, et al. 2003; Dauguet et al. 2007; Bastiani et al. 2012; Azadbakht et al. 2015). In combination with resting-state functional MRI, dMRI has proven successful for investigating cortical and subcortical organization in humans and nonhuman primates (Sallet et al. 2013; Chowdhury et al. 2013). dMRI results have also been used in patients to refine precise targeting of deep brain stimulation or brain lesion sites (Coenen et al. 2014; Saluja et al. 2020).

However, a number of recent studies (Thomas et al. 2014; Azadbakht et al. 2015; Donahue et al. 2016; Maier-Hein et al. 2017; Girard et al. 2020; Ambrosen et al. 2020) have investigated the limits of dMRI in terms of accurately visualizing and resolving brain structures by comparing neuronal pathways identified with in vivo tracers and tractography data from postmortem scans of monkey brains (Jbabdi, Lehman, et al. 2013). When looking at whether a range of area-to-area connections in the visual system were correctly captured by dMRI and tractography, Azadbakht et al. (2015) suggested a performance of about 75% for accurate detection of true interareal connectivity. However, this accuracy might have been inherently limited because those studies compared tracer and dMRI data from different brains. Others have compared tracer and tractography results in the same brains (Dyrby et al. 2007; Knösche et al. 2015) and found that the major fiber tracts could be well detected. These studies highlight the potential trade-off between sensitivity (detecting real connections) and specificity (rejecting false connections).

Ambrosen et al. (2020) also showed how scan parameters influence brain connectivity mapping. Using postmortem dMRI, they show that a lower image resolution (1.0 mm^3^ instead of 0.125 mm^3^) can improve connectivity mapping in agreement with tracer studies (Markov et al. 2014) as long as the image resolution is sufficient to spatially cover the pathway of interest. This supports our finding of in vivo dMRI performing well under some circumstances despite the lower image resolution compared to postmortem dMRI.

Rather than looking at the accuracy of detecting neuronal tracts between one brain area and another, the current study investigated how well dMRI and tractography could resolve the order of topographic connections between one brain area and another along a known anatomical tract. These ordered area-to-area connections form the basis of functional retinotopic maps in the visual system, to which we compared our structural results. Detection of these topographical relationships is a more demanding test for dMRI and requires a higher level of precision.

The focus on intra-area topography for this study led to particular choices for the implementation of tractography and analysis, designed to increase sensitivity and overcome potential nonlinear biases in the tractography (Jones 2010; Jones et al. 2013, Liptrot et al. 2014), which may be caused by the different sizes of and distances to target structures, in particular for central and peripheral V1. We normalized streamline incidence maps using PDFs, which helped to detect relatively weaker signals yielding fewer streamlines. We boosted overall streamline numbers by using extended target masks generated with a method similar to ICE-T by Liptrot et al. (2014). This was particularly important for the detection of the smaller cortico-cortical tracts. We carried out controls to show that—at least in the combinations used—these analysis choices did not fundamentally change the underlying topography result for the strong geniculo-cortical connectivity. Another important aspect is also that we constrained our analysis in ways that would increase specificity. We used exclusion masks to prevent impossible connections, for instance, across sulci and through ventricles. We had well defined seeds and targets, which were precisely checked and aligned to individual brains.

One surprise was therefore the failure to predict consistent V5/MT elevation maps of the superior–inferior division. This might be due to the functional arrangements of the visual areas in this location. Kolster et al. (2009, 2014) suggested that a cluster of areas in the STS, including area V5/MT, are organized around a common cortical point that represents the fovea, with eccentricity mapped concentrically and elevation mapped radially. Therefore, at its ventral margin, V5/MT neighbors a mirror reversal of its visual field representation in area FST. We found it challenging to define the ventral and dorsal borders of the V5/MT mask, and the standard atlas registration and the histological myelin borders derived from individuals’ histological sections were often in disagreement by a few millimeters. Neither approach to defining the V5/MT borders yielded consistently accurate results on a case-by-case basis (Large et al. 2016). Another potential explanation for this inconsistency might lie in the gyral bias when tracking fibers in thin gyral blades (Cottaar et al. 2021), as there is one posterior to the STS. But this does not straightforwardly explain the differences in success of predicting the two map dimensions (central-peripheral vs. superior–inferior). Overall, we were mostly successful in demonstrating the LGN maps and most V5/MT eccentricity maps, but the consistency of the V5/MT elevation maps was less reliable.

Strong and ordered connections between LGN and V1 clearly aided our consistent predictions of LGN topography postmortem and in vivo. Along the tracts, we observed regions of high streamline density that maintained the same topographic organization between LGN and V1. In particular, the retinotopic elevation mapping in LGN was evident well into the white matter, suggesting that the axons are organized the same way. Topographic organization of thalamic fibers has been observed in rats (Lozsádi et al. 1996; Molnár et al. 1998) and cats (Nelson and LeVay 1985), but some of these studies also indicated crossing over of fibers in the geniculo-cortical pathway, which is required to connect one topographic map to the other (Adams et al. 1997). Connolly and Van Essen (1984) predicted such a cross-over for one map axis in monkeys. Given the dorsal-ventral segregation of inferior and superior streamlines in our data and the slightly worse result for the central-peripheral visual topography prediction, we suggest that the map inversion occurs about the central-peripheral axis.

The current study provides evidence that we can successfully use the combination of diffusion-weighted imaging and probabilistic tractography to predict accurately ordered functional maps within a brain structure based on its structural connectivity with another ordered map. In patients, this approach could be used to predict, for instance, visual field damage after a stroke and the potential for recovery of function through connections with other structures. We propose DWI sequences and constraints for analysis protocols, including the use of white matter extended targets and PDF normalization. It will be particularly exciting to transfer this methodology to ordered brain maps and pathways other than those based on retinotopy. Potential structural relationships and target maps include the saliency map in the superior colliculus, the priority map in frontal eye fields, feature maps in V4, and saccadic maps in the posterior parietal cortex (Moore and Armstrong 2003; Schluppeck et al. 2005; Hafed and Krauzlis, 2008; Veale et al. 2017). Our cortico-cortical results (and the specific failures) suggest that area boundaries are a critical issue to get right.

## Conclusion

In conclusion, we find that dMRI with appropriate, well-constrained, probabilistic tractography analysis is sensitive and specific enough to resolve topographic mapping in thalamo-cortical connections, both postmortem and in vivo. The same is true to a somewhat lesser extent for cortico-cortical connections. These conclusions hold at the population level. With strong and well-ordered connections, it should be possible to compare the topography of connections in two groups of individuals, for example, in the comparison of clinical populations against a group of neurotypical subjects. Identification of individual differences in topography on a case-by-case basis for in vivo measurements might be achievable but has to be established for each specific topographic map and connection across a population first.

## Supplementary Material

Supplementary_Fig_1_revised_bhab364

Supplementary_Fig_2_revised_bhab364

Supplementary_Fig_3_revised_bhab364

Supplementary_Fig_4_revised_bhab364

Supplementary_Table_1_bhab364

Supplementary_Table_2_bhab364

## Notes

The authors would like to thank the University of Oxford’s BMS and veterinary staff and the DPAG workshop for expert technical support. *Conflict of Interest*: None declared.
