# Supplementary material for: Intra-Areal Visual Topography in Primate Brains Mapped with Probabilistic Tractography of Diffusion-Weighted Imaging
Source: Cereb Cortex. 2021 Nov 3;32(12):2555–74. doi: 10.1093/cercor/bhab364 (PMC9201591; doi:10.1093/cercor/bhab364)
Supplement: Supplementary_Fig_1_revised_bhab364 [file supplementary_fig_1_revised_bhab364.pdf]

Supplementary Fig. 1. Evaluating tractography parameters in one *post mortem* hemisphere.

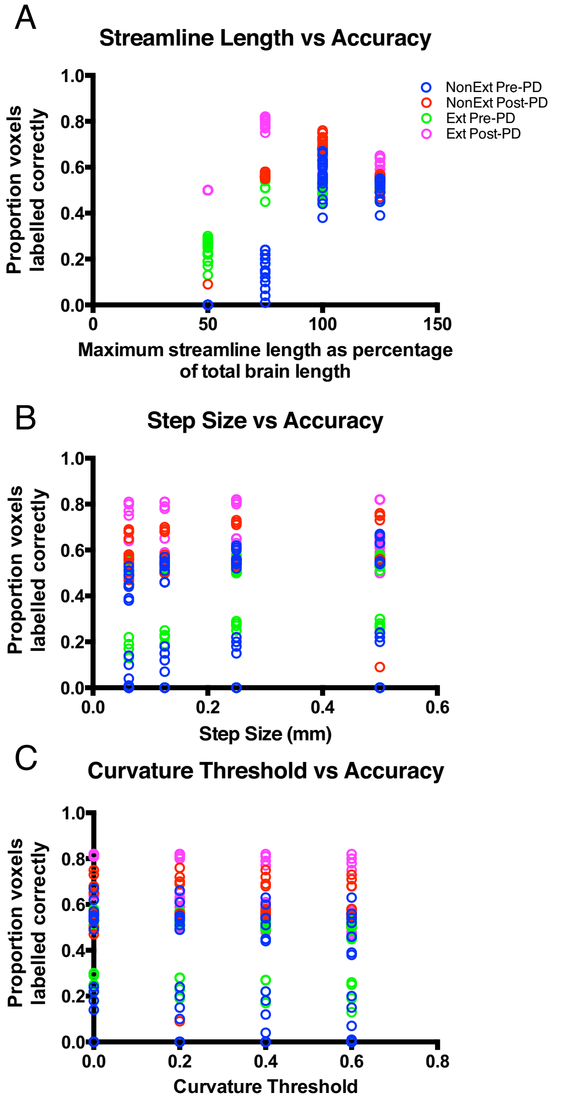

Scatter plot of the proportion of voxels correctly labelled with regard to the LGN electrophysiological atlas for every tested parameter combination, grouped by (**A**) maximum streamline length, (**B**) step length, and (**C**) curvature threshold. The results indicate that for the ranges tested, the highest accuracy can be achieved with a total maximum streamline length of 75% of brain length, 0.5 mm step length (= voxel size) and 0.0 curvature threshold (±90°) – in the context of extended target masks (“Ext”) and PDF (“Post-PD”), which were also used in this study.

Separately, we confirmed that this selection of parameters (streamline lengths 75%, step length = voxel side length [*post mortem*: 0.5 mm, *in vivo*: 1 mm], curvature

threshold 0) also maximised the number of streamlines that reached the central visual field representation of V1 from cortical area V5/MT (data not shown).
