# Supplementary material for: Intra-Areal Visual Topography in Primate Brains Mapped with Probabilistic Tractography of Diffusion-Weighted Imaging
Source: Cereb Cortex. 2021 Nov 3;32(12):2555–74. doi: 10.1093/cercor/bhab364 (PMC9201591; doi:10.1093/cercor/bhab364)
Supplement: Supplementary_Fig_2_revised_bhab364 [file supplementary_fig_2_revised_bhab364.pdf]

Supplementary Fig. 2. Example overlay of neurophysiological LGN atlas over the predicted map.

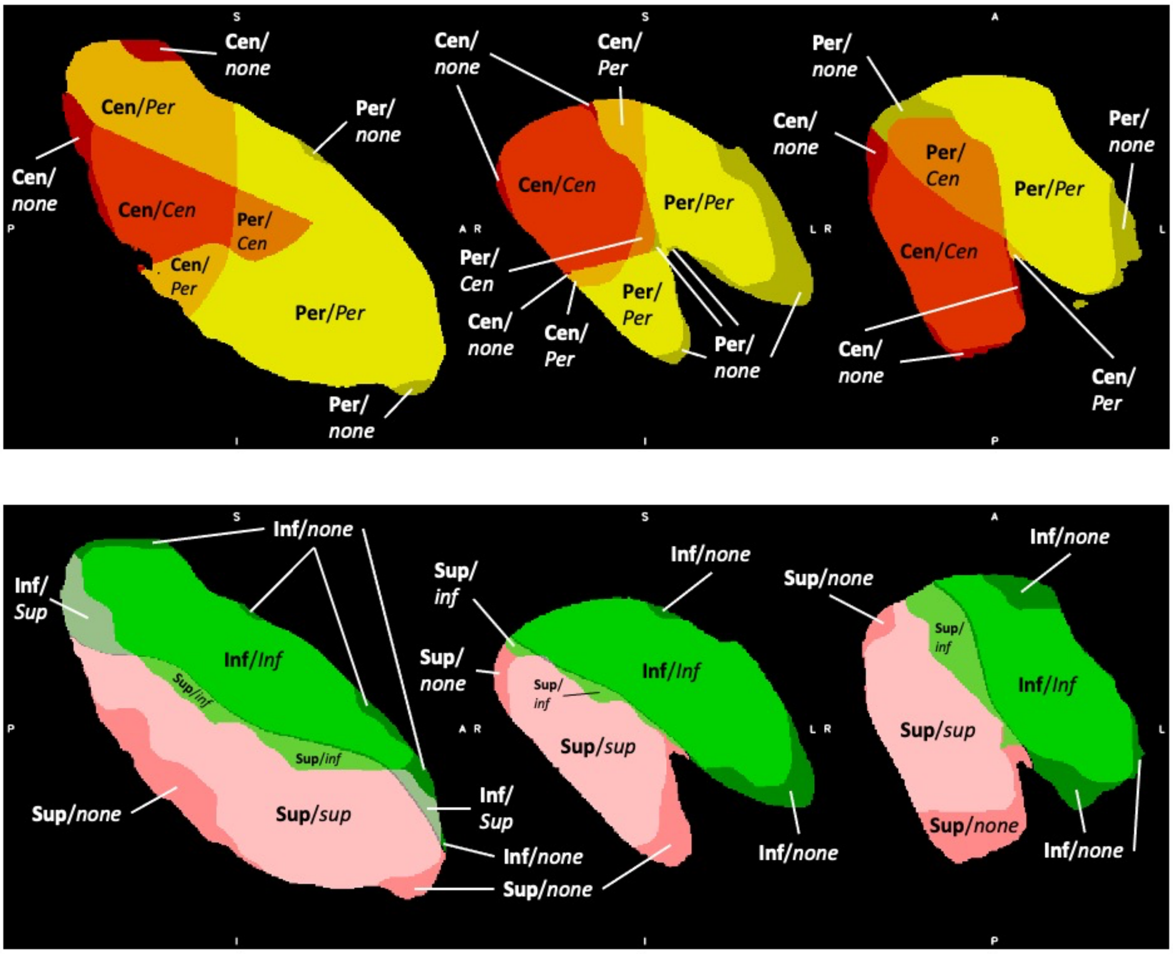

The figure shows two examples of the registration of the predicted topographical maps to the neurophysiological atlas of the LGN (Erwin et al. 1999). We show a parasagittal, coronal and horizontal section, each near the middle of the LGN. The central-peripheral segmentation (M126 left hemisphere; TOP) is somewhat poorer than the superior-inferior (M130 left hemisphere; BOTTOM). Topography of overlapping regions is described with this pattern **<Atlas>**/*<Prediction>*. “None” refers to areas where no MRI data was matched to the atlas after transformation.
