# Supplementary material for: Intra-Areal Visual Topography in Primate Brains Mapped with Probabilistic Tractography of Diffusion-Weighted Imaging
Source: Cereb Cortex. 2021 Nov 3;32(12):2555–74. doi: 10.1093/cercor/bhab364 (PMC9201591; doi:10.1093/cercor/bhab364)
Supplement: Supplementary_Fig_3_revised_bhab364 [file supplementary_fig_3_revised_bhab364.pdf]

Tang-Wright, Smith, et al. “Intra-areal visual topography in primate brains mapped with probabilistic tractography of diffusion-weighted imaging”

Supplementary Fig. 3. Dice Coefficient

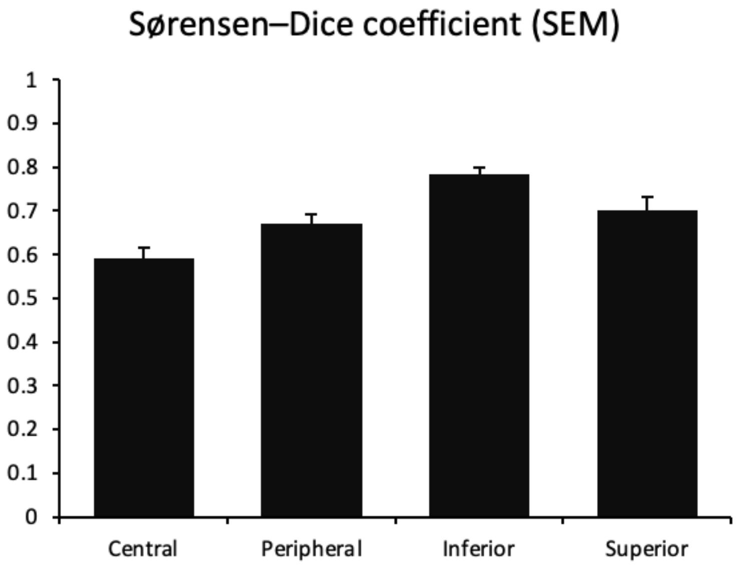

The graph plots the Sørensen-Dice coefficient to gauge the similarity between the topographic map representations we predicted from tractography of the DWI-data and the neurophysiological atlas of the LGN (Erwin et al. (1999)). The results are comparable to those shown in the main paper for our calculations of % correct (see Figure 6).
