# Supplementary material for: Intra-Areal Visual Topography in Primate Brains Mapped with Probabilistic Tractography of Diffusion-Weighted Imaging
Source: Cereb Cortex. 2021 Nov 3;32(12):2555–74. doi: 10.1093/cercor/bhab364 (PMC9201591; doi:10.1093/cercor/bhab364)
Supplement: Supplementary_Fig_4_revised_bhab364 [file supplementary_fig_4_revised_bhab364.pdf]

Tang-Wright, Smith, et al. “Intra-areal visual topography in primate brains mapped with probabilistic tractography of diffusion-weighted imaging”

Supplementary Fig. 4. Crossing fibres

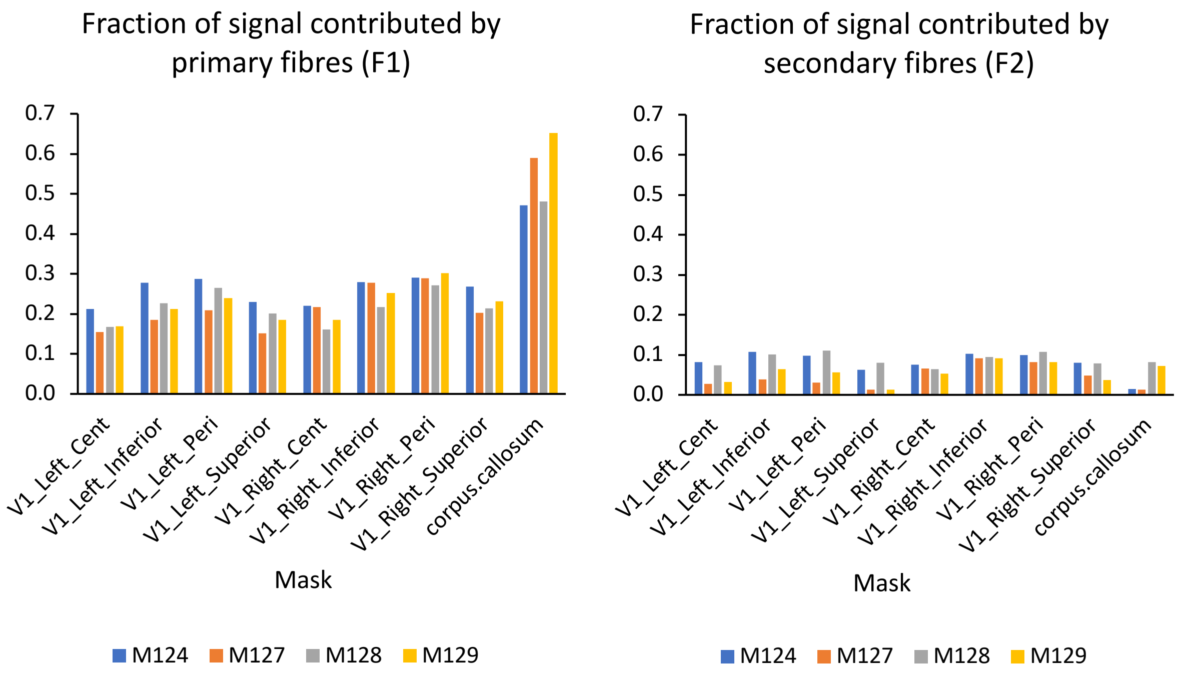

To further assess the quality of our *in vivo* data, which would be the data most relevant to clinical populations, we also compared anisotropic volume fraction contributed by primary and secondary fibers in our white matter tracts between LGN and V1. These were calculated by bedpostX and compared to the data from the corpus callosum from the same brains. The data show that the dMRI signal is dominated by the primary tract.
