# Supplementary material for: Intra-Areal Visual Topography in Primate Brains Mapped with Probabilistic Tractography of Diffusion-Weighted Imaging
Source: Cereb Cortex. 2021 Nov 3;32(12):2555–74. doi: 10.1093/cercor/bhab364 (PMC9201591; doi:10.1093/cercor/bhab364)
Supplement: Supplementary_Table_1_bhab364 [file supplementary_table_1_bhab364.pdf]

Tang-Wright, Smith, et al. "Intra-areal visual topography in primate brains mapped with probabilistic tractography of diffusion-weighted imaging"

Supplementary Table 1

	Central	Peripheral	Inferior	Superior
V1 (extended)	58.1 (8.8)	77.1 (10.7)	92.0 (4.7)	75.2 (13.0)
V1 (GM only)	37.2 (13.7)	94.9 (8.5)	84.6 (7.8)	72.5 (16.1)
Shorter maximum streamline length	56.3 (7.0)	95.4 (5.1)	91.9 (5.3)	70.3 (13.6)

	Cen & Per	Inf & Sup	Total
V1 (extended)	67.7 (8.8)	83.1 (8.2)	75.3 (11.5)
V1 (GM only)	65.7 (8.9)	78.6 (13.8)	72.3 (24.9)
Shorter maximum streamline length	75.8 (20.8)	81.1 (15.0)	78.5 (18.1)

We calculated the average percentage correct (standard deviation) for the predicted LGN topography (based on dMRI and tractography) of all 12 hemispheres in our data set with respect to the visual field regions as identified in previous neurophysiological data (LGN atlas published by Erwin et al. 1999). We compared these results for three different tractography sessions obtained varying different parameters: extended V1 target masks ("extended") – as used in this paper, V1 cortical target masks only (grey matter "(GM) only"), a maximum streamline length of 66% of total brain length instead of otherwise 75% ("shorter maximum streamline length"). To compare individual results, we defined the significance of paired t-tests using Bonferroni correction for 12 comparisons (significance level $\alpha = 0.05/12 = 4.167\times10^{-3}$).

When the target masks were confined to V1 grey matter ("V1 GM only"), the number of streamlines that found a target (mean = 1,567,759 [SD = 2,388,809]) was much lower compared with the extended target mask (mean = 47,067,839 [22,352,858]; paired Wilcoxon signed rank test, $Z = 6.03$, $p < 0.001$). While predicted

topographic maps were no less accurate overall (Tukey-Kramer test, p = 0.634), the variance increased (Levene’s test, $p < 0.002$) and the accuracy of the predicted topography (as used in the paper) varied with visual eccentricity: Map predictions were better for peripheral LGN (paired t-test, p < 0.001) and worse for central LGN (paired t-test, p < 0.003). In contrast, the predicted elevation maps were just as accurate (paired t-test, p = 0.476).

We used a maximum streamline length 75% of the brain’s length (this was optimal in a pilot brain, see Methods and Supplementary Fig. 1). We also tested here a more conservative threshold of 66% (‘Shorter maximum streamline length’). Neither the overall accuracy (Tukey-Kramer test, p = 0.743) nor reliability (Levene’s test, p = 0.268) of topographic prediction was affected. However, peripheral LGN was better predicted (paired t-test, p < 0.001), but the others were no different (p > 0.017).
