# Supplementary material for: Intra-Areal Visual Topography in Primate Brains Mapped with Probabilistic Tractography of Diffusion-Weighted Imaging
Source: Cereb Cortex. 2021 Nov 3;32(12):2555–74. doi: 10.1093/cercor/bhab364 (PMC9201591; doi:10.1093/cercor/bhab364)
Supplement: Supplementary_Table_2_bhab364 [file supplementary_table_2_bhab364.pdf]

Supplementary Table 2. FSL parameter settings used in this study.

**Parameter**	**Setting**
Loopcheck	Yes
Use modified Euler streaming	Yes
Use anisotropy to constrain tracking	No
Use distance correction	No
Subsidiary fibre volume fraction threshold	0.01
Minimum length threshold (mm)	0.0
Seed sphere sampling (mm)	0.0
